# Editorial: Fungal Respiratory Infections in Cystic Fibrosis

**DOI:** 10.3389/fcimb.2021.800847

**Published:** 2021-11-09

**Authors:** Nicolas Papon, Andrew Mark Borman, Wieland Meyer, Jean-Philippe Bouchara

**Affiliations:** ^1^ Univ Angers, Univ Brest, GEIHP, SFR ICAT, Angers, France; ^2^ UK National Mycology Reference Laboratory, United Kingdom Health Security Agency South-West, Bristol, United Kingdom; ^3^ Medical Research Council Centre for Medical Mycology (MRC CMM), University of Exeter, Exeter, United Kingdom; ^4^ Sydney Institute for Infectious Diseases, The University of Sydney, Sydney, NSW, Australia; ^5^ Molecular Mycology Research Laboratory, Centre for Infectious Diseases and Microbiology, Westmead Clinical School, Sydney Medical School, Faculty of Medicine and Health, The University of Sydney, Westmead, Sydney, NSW, Australia; ^6^ Westmead Hospital (Research and Education Network), Westmead, NSW, Australia; ^7^ Westmead Institute for Medical Research, Westmead, NSW, Australia; ^8^ Curtin Medical School, Curtin University, Perth, WA, Australia

**Keywords:** cystic fibrosis, yeasts, molds, infections, epidemiology, diagnostics, prophylaxis

Cystic fibrosis (CF), which predominantly affects Caucasian populations (Europe, Australia, North of America), is an inherited autosomal recessive genetic disease caused by mutations in the *CFTR* (Cystic Fibrosis Transmembrane conductance Regulator) gene that encodes a chloride channel located at the apical surface of numerous epithelial cell types. Many organs are therefore affected by this disease, but the outcome essentially depends on the extent of pulmonary involvement and damage. Indeed, mutations in the *CFTR* gene result, in the respiratory tract, in the thickening of the bronchial mucus and impairment of mucociliary clearance that together allow the entrapment of inhaled bacteria and desiccated yeast cells and filamentous fungal spores and provide a suitable environment for their proliferation. Thus the respiratory tract of CF patients often becomes colonized by various microorganisms, causing recurrent pulmonary exacerbations, which lead to a persistent inflammatory reaction and progressive deterioration of lung function. Bacteria, notably *Pseudomonas aeruginosa*, are the major cause of these infections. As a consequence, special attention has been paid to the prevention and treatment of bacterial infections. Progress in this field, including advances in the diagnosis of the disease as well as in the nutritional status of the patients, have progressively led to a significant increase in life expectancy. However, in addition to bacteria, many fungal species can also colonize the CF lung, potentially leading to respiratory infections, which increase in frequency in parallel with the increase in life expectancy. Although still controversial, there is now accumulating evidence that chronic colonization of the airways by fungi contributes to clinical or functional pulmonary deterioration. Even more problematic, in patients undergoing lung or heart/lung transplantation, this fungal colonization of the airways may lead to severe and often fatal disseminated infections because of the ability of these thermotolerant fungi to rapidly disseminate in a susceptible host and, for some of these pathogens, because of their low innate susceptibility or frank resistance to current antifungal agents.

Among the prominent fungal species colonizing the CF airways, *Candida albicans* for yeasts and *Aspergillus fumigatus* for filamentous fungi predominate. However, other fungi are increasingly reported in this context, including *Scedosporium* species, *Aspergillus terreus, Exophiala dermatitidis*, species in the *Rasamsonia argillacea* complex, and *Lomentospora prolificans*. In addition, pioneering studies of the microbiota have suggested an even greater diversity of the fungal flora colonizing the CF airways. Unfortunately, unlike bacterial infections, our knowledge about the epidemiology and pathogenesis of fungal respiratory infections in CF remains scarce and sustained research on this topic is needed. Therefore, we are delighted to introduce the Research *Topic “Fungal Respiratory Infections in Cystic Fibrosis”* in Frontiers in Cellular and Infection Microbiology to highlight some tremendous advances in this field.

For this Research Topic, we have collected twelve articles, including eleven original research studies and one review.

First, Bonnet et al. provided an excellent compilation of recent data about *Pneumocystis jirovecii* in CF. This review clearly demonstrates that knowledge in the field is still fragmented, and this topic remains an open area of investigation. Of importance, a multicenter prospective study using standardized methods for *P. jirovecii* screening in CF patients including lung transplant recipients, and MLST/NGS analysis of the *P. jirovecii* genome combined with patient-age stratification, CFTR genotyping, and microbiome/mycobiome analysis is warranted and long overdue.

Alongside this review, a series of three original articles deal with various aspects of the biological surveillance of fungal respiratory infections in the context of CF. First, Grehn et al. provide evidence that frequent contacts with pets may be a risk factor for allergic bronchopulmonary aspergillosis (ABPA) in patients with CF, suggesting that such information should be requested when questioning the patient about social history and lifestyle, especially for *A. fumigatus*-sensitized patients or patients with recurrent ABPA. In another original study, Grehn et al. show that urban life should be considered as a risk factor for airway colonization by *A. fumigatus*. This study has the merit of making us aware of the influence of environmental factors on the clinical course of CF patients, and this information also should be collected during patient interviews. Finally, this series of articles ends with the report from Guegan et al. that underscores a huge variability in the degree of azole resistance in *A. fumigatus* depending on the azole drug, the patient origin, and the clinical setting, but also among different isolates from a single patient. In addition to epidemiological data, this study highlights the need for a global reflection that must be engaged towards changing our routine procedures for mycological examination of respiratory secretions from CF patients to include systematic *in vitro* susceptibility testing of *A. fumigatus* isolates to azole antifungals.

Other breakthroughs introduced in this Research Topic concern the diagnosis of fungal respiratory infections in CF patients. For example, the report from Eschenhagen et al. demonstrates that the presence of serum-specific anti-*A. fumigatus* IgG in CF patients may be a useful marker for acute ABPA and *A. fumigatus* pneumonia, but not for *A. fumigatus* bronchitis, although it should only be interpreted together with other biological markers. A standardized multicenter longitudinal study should be conducted on a larger CF cohort since better knowledge of underlying immunological mechanisms may improve its clinical utility. In addition, Patel et al. investigated the relationships between some airway biomarkers, including the leukocyte differential cell count and the level of neutrophil elastase, interleukin-8, galactomannan, and tumor necrosis factor receptor type 2 in sputum or bronchoalveolar lavage fluid samples, and fungal culture positivity. Fungal culture positivity was found to be associated with exposure to indoor molds, bronchiectasis, and diminished lung function, but no relationship was seen with the different biomarkers that were evaluated. In their report, Martin-Souto et al. have selected a crude antigenic extract of *S. boydii* to detect IgG antibodies directed towards *Scedosporium* spp. and *L. prolificans* in sera from CF patients. An ELISA test was developed, showing very high sensitivity and specificity, which therefore may improve the serodiagnosis of *Scedosporium/Lomentospora* infections. This serological method may also be interesting for monitoring the evolution of infection and to monitor the effectiveness of antifungal interventions.

In this Research Topic, we are also delighted to introduce a report from Currie et al., which demonstrates that CFTR modulators may have additional immunomodulatory benefits to prevent or treat *Aspergillus*-induced inflammation in CF. In this study, it is particularly intriguing to observe the comparable effects of CFTR modulators in phagocytes from CF patients and from control individuals, thus raising important questions about their exact mechanism of action.

Another series of investigations compiled in this Research Topic provides interesting information about the pathogenesis of fungal respiratory infections in CF with special emphasis on microbial interactions that may occur in the CF lung. First, Roisin et al. shed light on microbial interactions modulating the susceptibility of pathogens to antimicrobial drugs in polymicrobial biofilms. More specifically, they show that *Stenotrophomonas maltophilia* increased, in these biofilms, the susceptibility of *A. fumigatus* to amphotericin B, whereas *A. fumigatus* protected *S. maltophilia* from levofloxacin. This opens new windows for analyzing in the near future the antimicrobial susceptibility of pathogens in polymicrobial biofilms and to defining the mechanisms underlying such changes in their susceptibility. In the same area, Bertulino de Oliveira et al. have tested the effect of peptidorhamnomannans (PRMs) extracted from *L. prolificans*, *Scedosporium apiospermum, Scedosporium boydii*, and *Scedosporium aurantiacum* on bacterial species relevant to CF (e.g. *P. aeruginosa*, *B. cepacia*, methicillin-resistant *S. aureus*, and *Escherichia coli*). Interestingly, their data suggest that PRMs from *Scedosporium* and *Lomentospora* cell surfaces may play an important role in fungal colonization by disrupting some bacterial populations. In addition, Gonçalves de Almeida et al. have explored the lung microbiome of three young CF patients colonized by fungi. They found a personal signature with low variation in the microbiome across pulmonary exacerbations and a core set of virulence factors and antibiotic resistance genes. This study is important as understanding the microbial community and its interactions is crucial to improving therapeutic interventions by avoiding deleterious therapies that inadvertently restructure the pathogenic microbiota. Future studies focusing on the influence of fungi on bacterial diversity and microbial interactions in the CF microbiome will be welcome to fulfill the huge knowledge gap concerning the influence of fungi on the evolution of CF pulmonary disease. Last but not least, Le Govic et al. have used both functional genomics and metabolomics approaches to demonstrate that the *S. apiospermum sidD* gene drives the synthesis of a unique extracellular siderophore, *N* ^α^-methylcoprogen B, that was found to be essential for fungal growth and virulence. This secondary metabolite seems important for iron acquisition from pyoverdine, which might explain the apparent antagonism between *S. apiospermum* and *P. aeruginosa* within the CF lung.

Overall, this series of reports show that sustained research projects aimed at deciphering the epidemiology and pathogenesis of fungal respiratory infections in the context of CF are now ongoing worldwide. This is, of course, an essential prerequisite for faster progress in the prevention, diagnostics (notably for differentiating transient carriage and chronic colonization of the airways), and treatment of these life-threatening infections.

## Author Contributions

All authors contributed to this editorial. NP and J-PB wrote the initial manuscript. AB and WM revised the manuscript, and all authors approved the submitted version.

## Conflict of Interest

The authors declare that the research was conducted in the absence of any commercial or financial relationships that could be construed as a potential conflict of interest.

## Publisher’s Note

All claims expressed in this article are solely those of the authors and do not necessarily represent those of their affiliated organizations, or those of the publisher, the editors and the reviewers. Any product that may be evaluated in this article, or claim that may be made by its manufacturer, is not guaranteed or endorsed by the publisher.

